# The Coronal Tooth Fractures: Preliminary Evaluation of a Three-Year Follow-Up of the Anterior Teeth Direct Fragment Reattachment Technique Without Additional Preparation

**DOI:** 10.2174/1874210601711010266

**Published:** 2017-06-30

**Authors:** Lo Giudice G, Alibrandi A., Lipari F, Lizio A, Lauritano F, Cervino G, Cicciù M

**Affiliations:** 1 Department of Clinical and Experimental Medicine, Messina University, Cannizzaro, Messina, Italy; 2 Department. of Economics, Statistics, Mathematics and Sociology, Messina University, Messina,Italy; 3 Department of Biomedical and Dental Sciences and Morphofunctional Imaging, Messina University, AOU Policlinico “G. Martino” Messina, Italy

**Keywords:** Adhesive technique, Crown fracture, Dental trauma, No preparation, Reattachment, Tooth fragment

## Abstract

**Objective:**

The aim of this research is to describe and to analyse the long-term results and the clinical steps of direct fragment reattachment technique with no additional tooth preparation, used to treat crown fracture. This technique achieves the clinical success, combining satisfactory aesthetic and functional results with a minimally invasive approach.

**Methods:**

The 3 years follow-up included 9 patients (5 males, 4 females) with coronal fracture. In all the cases the fragment was available and intact. The authors illustrate the adhesive procedure used. Under local anaesthesia and after positioning the rubber dam, both the tooth and the fragment surface were etched, rinsed and applied by the adhesive system in order to obtain the retention of the fractured part to the tooth without additional tooth preparation or resin cement.

**Results:**

The statistical analysis shows the good performances of direct fragment reattachment technique. After 36 months, in 22.2% of the cases, the detachment was observed of the bonded fragment and in 11.1% of patients, complications were recorded.

**Conclusion:**

Our clinical experience shows how the ultra-conservative procedure used is fast, easy and offers a long term predictability; it also allows good functional and aesthetic outcomes.

## 1 INTRODUCTION

The maxillofacial traumatic injuries often occur in association with dental trauma. An epidemiologic study of Gassner *et al*. [1] revealed an incidence of 48.25% for dental injuries in all facial trauma. According to this study, the literature states a mean prevalence of 50% [2, 3]. Moreover, the severity of dental traumatic damage, in patients who have a combination of dental injury and majormaxill ofacial injury, is usually different from those in which a simple dental trauma occurs [2].

The type of dental primary dentition most frequent damage is the subluxation, the uncomplicated crown fracture usually occurring in permanent teeth [4].

The literature recognizes that incisors are frequently affected from coronal fractures (18-22% of all dental traumas), with the prevalence of 96% referred to central maxillary incisor [1, 5-7].

In this kind of fractures, the loss of mineral structure involves only the enamel or both the enamel and the dentin, without any pulp exposure.

Various techniques and materials have been proposed and used to restore enamel-dentin fractures, depending on different clinical situations.

The missing tooth portion can be restored by means of a direct restoration, using composite resins or indirectly choosing lab processed composite or ceramic inlays [8].

In the age of the minimal invasive and conservative dentistry, when a correctly preserved fractured portion is available, the adhesive reattachment to the residual tooth structure should represent the first treatment choice.

The clinical success of this procedure, that allows re-using the original fragment, is enhanced by the improvement, in reliability and effectiveness, of modern adhesive systems.The presence of supra gingival margins is a basic condition to perform the fragment reattachment.

In this case, the fractured surface is visible and, for this reason, easy to access [9]. Based on our experience, the fragment should be stored in a medium in order to avoid dehydration and possible discoloration. Some authors suggest storing it in physiological solution at 37° C. in a closed container [10].

The fragment reattachment technique has many advantages over traditional restorative procedures because it is more conservative and it offers the clinician the possibility to re-establish the contour, the architecture, and the tooth’s original brightness [11].

When the fracture is associated with pulp exposure, it is classified as a complicated fracture and an endodontic treatment can be considered before the direct fragment reattachment technique is performed. In this case, a careful evaluation of the biological width and a minimal invasive endodontic access must be performed in order to obtain a long-term clinical success.

Fragment retention is significantly correlated to the technique and to the restorative materials used for the reattachment treatment. A number of operative procedures have been reported in literature, from little or no additional tooth preparation to various preparation options such as: placement of a circumferential bevel, placement of an internal groove, placement of an external chamfer, use of a superficial over-contour of material on the fracture line [12].

The authors have evaluated the functional and aesthetic outcomes, the versatility and the long-term stability of a tooth fragment reattachment technique without additional tooth preparation used to restore crown fractures just using bonding agent.

## 2 MATERIALS AND METHODS

### 2.1 Study Design and Patients

A prospective clinical study was performed between 2007 and 2015 in Department of Dentistry at the University of Messina. The study included 9 patients (5 males, 4 females) with tooth coronal fracture. In all the cases, the fractured fragment was available and intact and there were not macroscopic losses of dental tissue at the tooth-fragment interface.

For each patient, the clinical evaluation was carried out during a 4 years observation period. The teeth involved showed no evidence of caries, pulpal pathology or periodontal lesions consequent to the dental injury. The clinical status of the injuries is summarized in (Table. **1**).

### 2.2 Operative Phase

The fractured portion, evaluated sufficiently intact and with adequate margins and structure, was disinfected with 0.2% chlorexidine and temporarily stored in physiological solution to obtain the hydration.

**Fig. (1):**
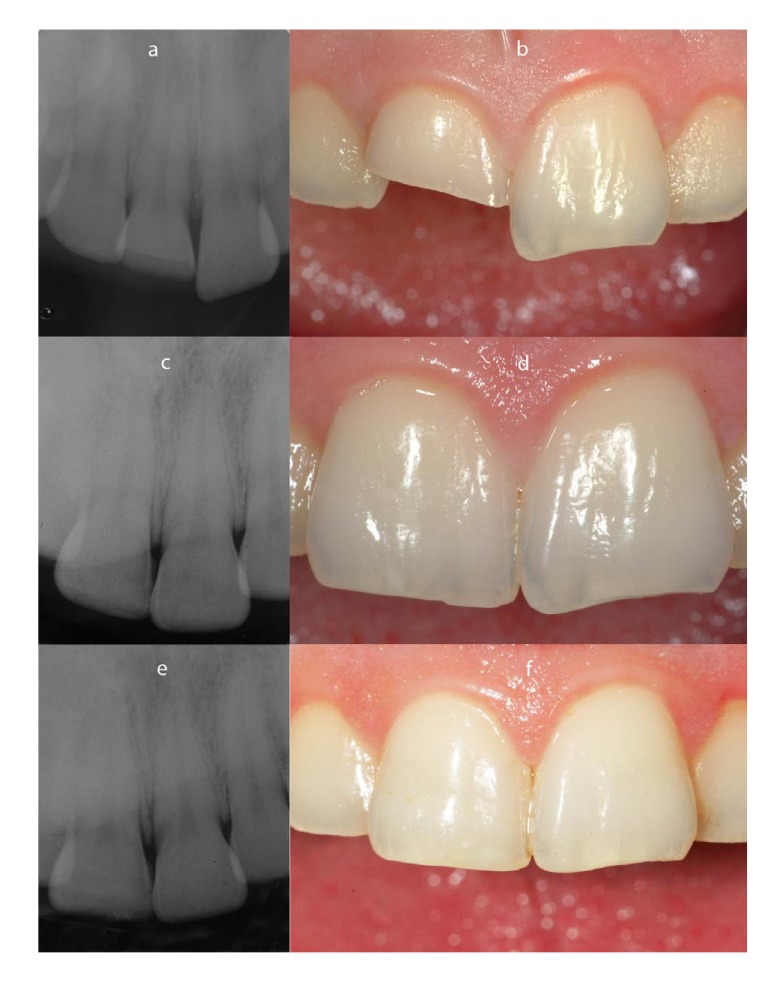
Patient # 3 (a - baseline rx control; b - fractured tooth; c - 12 months Rx control ; d - Clinical appearance after 12 months; e - 36 months Rx control; d - Clinical appearance after 36 months.

**Fig. (2):**
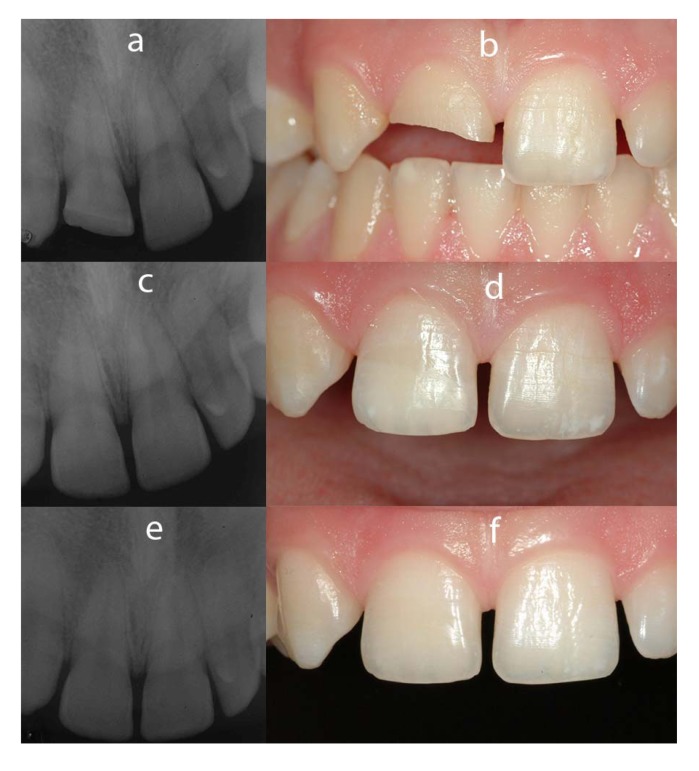
Patient #6 (a - fractured tooth; b - baseline rx control c - 12 months Rx control ; d - Clinical appearance after 12 months; e - 36 months Rx control; f - Clinical appearance after 36 months.

An accurate evaluation of the fractured tooth was performed Figs. (**1a**-**2a**). Vitality and mobility tests were useful to reveal a possible dislocation injury or the interruption of nerve and blood supply to the pulp. A periapical radiograph was performed (Figs. **1b**, **2b**, **1e**, **1f**, **2e**-**2f**).

The first step of the operative procedure, after local anaesthesia, was the isolation of the operating field with a rubber dam in order to avoid the negative effects of saliva and gingival crevicular fluid on the adhesive system.

Prior to the reattachment procedure, the fractured tooth was cleaned and polished and the fractured portion was “tried-in” many times attempting to adapt the fragment in the right position and to verify the absence of disruptions or defects between the remaining tooth structure and the fragment itself. To facilitate the fragment’s handling, it was fixed on its vestibular aspect to a holder with an adhesive tip (Pic-n-stick, Pulpdent Corp.).

**Fig. (3):**
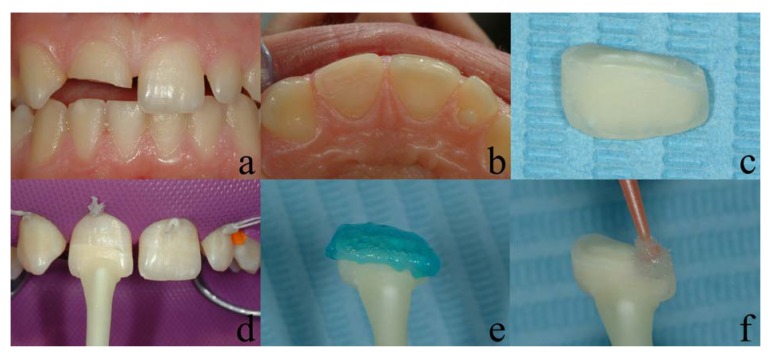
(a - buccal view; b - occlusal view; c - fractured fragment; d - fragment adaptation; e,f - adhesive procedures of the fragment).

Subsequently, the fragment was etched with 37% phosphoric acid gel and rinsed after 30 sec (30 for enamel, [15] for dentine) Fig. (**3**). The adhesive system was then applied on the etched surface and the fragment was kept away from light or heat sources till the reattachment phase (Fig. **3**).

**Fig. (4):**
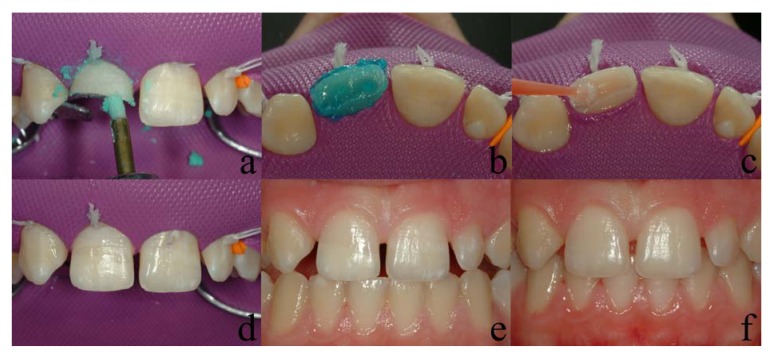
(a - cleansing; b - acid etching; c - application of adhesive agent; d - the finalized case; e -buccal view; f one - months postoperative view)

Also the remaining tooth structure was treated with an “etch and rinse” technique using a 3-step universal dental adhesive system (All-Bond BISCO) (Fig.**4**).

The fragment was thus placed in its proper position on the tooth under magnification (4X) to achieve the perfect fit between the two parts and only at this point, the bonding agent was photo-polymerized [13].

During the polymerization, it is essential that the fractured portion is not moved. The polymerization was carried out on both the labial and lingual aspects using a LED light (T-LED dna Anthos) with increasing light intensity (100 mW/cm^2^ for 10 sec; increasing intensity for 10 sec; 500 mW/cm^2^ for 20). Then the restored tooth was finished and polished using silicon points with a decreasing granulometry (HiLuster Plus Identoflex, KerrHawe) (Fig. **4**).

### 2.3 Follow-Up Parameters

The follow-up was performed at 12-36 months, both clinically and radiologically, to evaluate:

- Fragment position and stability
- Gingival swelling
- Presence of signs of endodontic and periapical pathology (response to vitality test,
  sensitivity to percussion, sinus tract formation, pulp canal obliteration, intactness of the lamina dura, apical radiolucency)
- Discoloration of the fragment or marginal pigmentation (Figs. **1b**, **1c**, **2b**, **2c**) (Table **2**).

### 2.4 Data Analysis

The examined variables are categorical and, so, are expressed as number and percentage. For data analysis, the non-parametric approach was used because of the low sample size.

Both for complications and for detachment (and their combination), the McNemar test was applied in order to assess the differences between paired proportions; in particular, we performed comparison between baseline *vs* 12 months Fig. (**2d**) and baseline *vs* 36 months (Fig. **1d**).

Statistical analyses were performed using SPSS 17.0 for Window package. *P*< 0,05 was considered to be statistically significant.

## 3 RESULTS

In the sample analyzed, the dental trauma was related in 22.2% of cases to a maxillofacial trauma. The coronal fractured tooth sample was composed by incisor (Superior: 6 centrals, 2 laterals; Inferior: 1 lateral).

In 55.5% of the patients, dentinal sensibility was referred, associated, in one case, with slight pulp exposure. The 44.4% of trauma was totally asymptomatic.

Within one year of the trauma, only one case of fragment detachment was reported; in the same case (11.1%), dentinal sensibility was present, with other teeth being totally asymptomatic.

In all the cases, no endodontic lesions were recorded. The teeth responded normally to vitality tests and were non-tender to pressure and percussion. The periapical radiograph showed no widening of the periodontal space and an intact lamina dura.

At 3-years follow-up, a new case of fragment detachment was recorded. In 11.1% of cases, marginal leakage with infiltration, associated to marginal discoloration was recorded. All teeth responded normally to vitality tests. When we compare compliance at baseline *vs* 12 months and 36 months, no difference exists *(p*=0.125 and 0.375, respectively).

Focusing our attention on the detachment, we found a significant change in comparison between baseline *vs* 12 months (*p*=0.008) and *vs* 36 months (*p*=0.016).

Finally, analysing compliances and detachment together, we found a significant change in comparison between baseline *vs* 12 months *(p*=0.008) and *vs* 36 months (*p*=0.031).

## 4 DISCUSSION

In our prospective clinical study, the crown fracture associated to maxillofacial trauma was observed in 22.2% of our patients.

H. Thore *et al*. Stated in a retrospective study on 389 patients with facial fracture referred an incidence of 16% of dental injuries. In a study by Robert Gassner *et al*., 1 in 6000 patients with facial injuries reported an overall incidence of 48.25%.

In our case series (uncomplicated fractures with intact margins of teeth and fragment), the most prevalent fracture site was the maxillary incisor region (88.8%).

Dentinal sensitivity was the most common complication (55.5%) with a single case of slight pulp exposure. In one patient, a subluxation was observed.

Our case series underlines the positive aspects of the reattachment technique used on fractured teeth.

The choice of the reattachment treatment without additional preparation was determined by the absence of macroscopic losses of dental hard tissue at the tooth-fragment interface.

Demarco *et al.* and Reis *et al*. stated the necessity of the application of a bevel, a chamfer or an over-contour to improve the fracture resistance after reattachment treatment [14, 11].

Another study by Reis *et al*. concluded that an internal groove and over-contouring determined a better resistance to fracture of reattached teeth [15]. The fracture resistance reached 90% of the control resistance and this finding could be related to the surface area of adhesion.

Wiegand *et al.* suggested the use of an internal groove when the residual dental structure and the fragment fit perfectly; otherwise a overcontour is advisable when there is a partial loss of hard tissue [16] .

However, some authors reported in clinical follow up that it is not necessary to use additional tooth preparations to ensure clinical success [16-21].

Reis *et al.* and Pusman stated that the amount of strength recovery needed for long-term fragment retention remains unknown, and suggested that fracture strengths as low as 50-60% may be sufficient for clinical success [14, 22].

Moreover, many studies performed on animal or human specimens have not found, at the moment, an agreement on the testing protocol as far as load speed, force direction and specimen preparation are concerned.

Therefore, there is no clear clinical evidence that any type of tooth preparation can really improve the fracture strength of the reattached fragment.

The long-term results and clinical observations show two cases of detachment observed at 12 months and 36 months follow-up respectively. In the last one, the detachment occurred after a new trauma.

The statistical analysis showed the significant data of the direct fragment reattachment technique during the observation time (12 months *p*=0.008; 36 months *p*=0.016).

According to Munksgaard et al., the primary cause of failure of the reattached tooth fragment is a new trauma or the use of the restored tooth with excessive masticatory forces [18].

The incidence of complication (sensitivity and marginal discolaration) after one and 3 years was 11.1%, with a reduction of the baseline values (66,6%).

In the patient with marginal discoloration (only 1 patient about 11.1%), periodontal inflammation was also observed due to poor hygiene. So no significant relationship of discoloration and periodontal disease has been recorded.

The improvement of generally evaluated compliances was substantial both after 12 months (*p*=0.008) and 36 months *(p*=0.031).

The results of long-term follow-up and clinical observations confirm, in our case series, that the reattachment technique is predictable and reliable and easy to reproduce.

## CONCLUSION

The direct fragment reattachment technique is an effective and an excellent alternative to direct and indirect restorations.

This reattachment technique, besides its aesthetic qualities (no composite restoration can accurately reproduce the optical properties and the characterizations of the dental structures), is faster to perform when compared to the direct restoration technique because it permits to avoid all the prosthetic steps related to the realization of a silicon matrix mandatory to correctly model the restoration palatal surface and to create a base for the subsequent composite’s layering.

The direct fragment reattachment technique compared to the prosthetic techniques (veneers and crowns), besides being more conservative, can produce immediate results without the need of various laboratory procedures, generating better patient compliance.

The good long term clinical results underline that how this technique is easy to standardize and perform, that it is inexpensive and that it allows a perfect aesthetic recovery and functional restoration of the fractured element, not underestimating the significant preservation of the dental structure.

When using these treatment procedures, a long-term fragment retention is achieved along with long term functional restoration exploiting a normo-functional compatible mechanical resistance, in accordance with the literature.

Moreover, a small pulp exposure is compatible with direct fragment reattachment technique that can preserve the tooth vitality eliminating the need of a endodontic therapy. This procedure is flexible and permits minimally invasive therapy taking advantage of dental adhesion and allowing the restored tooth to obtain a fracture resistance compatible to the functional stress of a sound tooth.

This therapy is particularly appropriate in case of associated maxillofacial trauma, because it is minimally invasive and fast and easy to perform.

The analysis of the clinical results exhibits the interesting aesthetic and functional significance of this technique confirming that the adhesive reattachment is the best treatment for an enamel-dentin fracture when the tooth fragment is available, sufficiently intact and well preserved.

## Figures and Tables

**Fig. (5):**
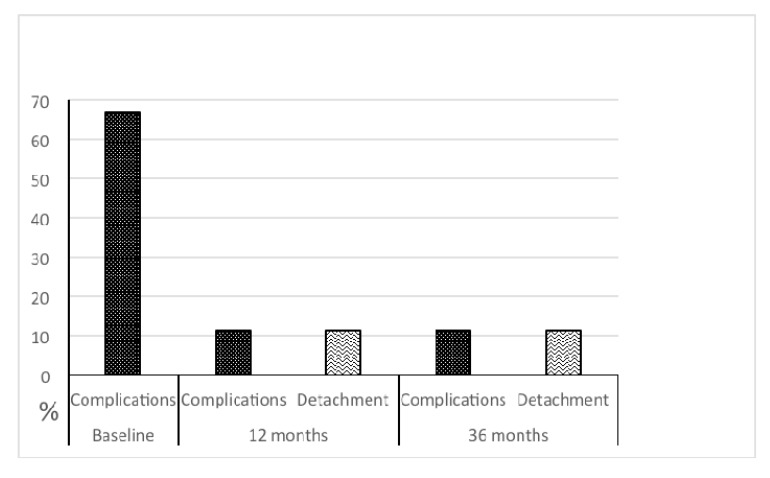
Shows the survival rate during the follow-up period.

**Table 1:** Baseline clinical status.

**Patient #**	**Gender**	**Teeth**	**Referred Symptoms**
1	M	2.1	Sensitivity
2	M	1.1	Sensitivity, Associated maxillofacial trauma
3	F	1.1	Absence of symptoms
4	M	2.2.	Slight pulp exposure, Sensitivity
5	F	3.2	Absence of symptoms, Associated maxillofacial trauma
6	M	1.1	Absence of symptoms
7	F	2.1	Sensitivity, subluxation
8	F	1.2	Absence of symptoms
9	M	1.1	Sensitivity

**Table 2:** Clinical reattachment follow-up after 12 and 36 months.

**Patient #**	**Clinical Status after 12 Months**	**Clinical and Radiological Status after 36 Months**
1	No pathological signs	No pathological signs
2	No pathological signs Periodontal phlogosis (poor oral hygene)	Marginal pigmentation
3	No pathological signs	No pathological signs
4	No pathological signs	Detachment of the fragment (another traumatic injury)
5	No pathological signs	No pathological signs
6	No pathological signs	No pathological signs
7	No pathological signs	No pathological signs
8	No pathological signs	No pathological signs
9	Sensitivity Detachment of the fragment	No pathological signs
